# Changes in patterns of eating habits and food intake during the first German COVID-19 lockdown: results of a cross-sectional online survey

**DOI:** 10.1007/s00394-022-02919-7

**Published:** 2022-06-27

**Authors:** Judith Bühlmeier, Stefanie Frölich, Christine Ludwig, Nadja Knoll-Pientka, Börge Schmidt, Manuel Föcker, Lars Libuda

**Affiliations:** 1 Department of Child and Adolescent Psychiatry, University Hospital Essen, University of Duisburg-Essen, Essen, Germany; 2 Faculty of Natural Sciences, Institute of Nutrition, Consumption and Health, Paderborn University, Paderborn, Germany; 3 Institute for Medical Informatics, Biometry and Epidemiology, University Hospital Essen, University of Duisburg-Essen, Essen, Germany; 4 LVR Clinic for Psychosomatic Medicine and Psychotherapy, University of Duisburg-Essen, University Hospital Essen, Essen, Germany; 5 Department of Child and Adolescent Psychiatry, University Hospital Münster, Münster, Germany; 6 Evangelisches Krankenhaus Düsseldorf, Children’s Hospital, Düsseldorf, Germany

**Keywords:** Coronavirus, Pandemic, Eating behavior, Dietary habits, Lifestyle

## Abstract

**Purpose:**

The COVID-19 pandemic and public measures have a direct impact on the nutrition situation; studies show changes in food consumption, eating behavior or body weight but complex pattern analyses of changes rarely exist.

**Methods:**

During the first German lockdown, a web-based survey was conducted among adults. It included 33 questions about changes in food intake, eating habits and physical activity, as well as anthropometrics and sociodemographic factors. Patterns of change were calculated based on changes in food intake and eating habits using two-step cluster analysis. To identify influencing factors for assignment to the patterns of change, binary logistic regression analyses were performed.

**Results:**

Data from 2103 participants (81% female, 40 ± 14 years) were considered for analysis. Increased stockpiling, cooking, and variation in preparation was reported by 50–70%. The constant pattern (C-P, 36%) reported little change besides the above. The health-oriented pattern (HO-P; 37%) reported eating more healthy foods, avoiding unhealthy foods, and eating less and less frequently. The emotional-driven pattern (ED-P; 28%) exhibits higher influence of emotions on eating behavior, less avoidance of unhealthy foods, and increased consumption of sweets, pastries, and alcohol. The odds of changing eating behavior either to HO-P or ED-P were higher in women, people with migration background, younger participants, and increased with BMI categories.

**Conclusion:**

Both, the ED-P and HO-P, exhibit distinctive reactions in eating habits and food intake when dealing with a distressing experience. In subgroups, these may lead to disturbances in eating behavior and increase the risk for eating disorders and obesity.

## Introduction

On March 11th, 2020, the World Health Organization declared the COVID-19 pandemic [1]. At this time, all German federal states had registered infections, first cases of death had been recorded [2] and the national spread of the virus took dynamic increases. Based on the acute risk assessment of the German Academia of Natural Science [3] from March 13th, the German government ordered several measures to control the virus spread and to reduce burden on health care systems to finally protect those at high risk (first German COVID-19 lockdown). The measures included closings of daycare, educational and cultural institutions, sport facilities, gastronomy, and shops. Where possible, employees and employers were strongly encouraged to switch to working from home. Borders were partially closed and travelling restricted [2]. Finally, on March 22nd, a limitation of private contacts in public spaces to two persons and minimum distances for social contacts were enacted [4].

Impacts on mental health caused by such lockdowns were detected early on [5]. Findings from Germany [6, 7] and many other countries [8] revealed high levels of psychological distress as well as symptoms of anxiety and depression in large parts of the general population. It is well accepted that food intake may serve as a coping strategy in dealing with uncomfortable feelings and difficult aspects of life in affluent societies [9, 10]. Besides longtime lockdown, perceived nutritional insecurity and confinement force people to develop new routines of daily activities such as meal planning. Thus, concerns were raised that the pandemic may indirectly facilitate unhealthy behaviors and increase the risk of non-communicable diseases [11]. A large variety of studies addressing dietary changes from all over the world has been released. A recent systematic review considering results from 95 studies found the majority of studies reporting an overall increase in snacking and amount of food eaten (87 and 81% of studies). Although studies reveal differing directions of changes in food group consumption, increased intakes of fruit and vegetables, but also of unhealthy foods (e.g. processed foods) are more pronounced than decreased intakes (e.g. 59% vs. 31% for unhealthy foods) [12]. People who gained weight during pandemic lockdowns, reported greater overall food intakes and intakes of energy-dense foods, less diet quality, increased snacking behavior as well as eating as a response to negative emotions [13–15]. When studying specific appetitive and cognitive traits and their association to overeating of energy-dense, savory foods, however, Buckland et al. found low craving control repeatedly to be the main characteristic of overconsumption and no associations to food responsiveness, enjoyment of food, emotional undereating, emotional overeating and satiety responsiveness [16, 17].

Despite heterogeneous reactions, the majority of studies present their results as a summary of their entire study population, e.g. overall increases/decreases/constancy in food intake/food group consumption/eating behaviors. Furthermore, these studies mainly contributed to the current evidence by addressing either food intake or traits of eating behavior. With the presented study, we aimed to explore dietary reactions to the COVID lockdown by cluster analysis and thus, to identify subgroups with similar patterns of change from a large-scaled population-based online survey conducted during the first German lockdown. Therefore, we integrated both, changes in eating habits and food intake. In exploratory analyses, we additionally assessed changes in body weight, physical activity, and potential stresses (latter one is not reported here) due to the lockdown for cluster characterization. The presented findings may provide deeper insights regarding complex changes in eating patterns of selective subgroups after a disrupting life event and thus, generate information for more targeted healthcare prevention.

## Methods

A German online survey was prepared using the web-based software EFS Survey (academic program “Unipark”, provided by Questback GmbH, Cologne, Germany). The link to the survey was shared on several platforms (e.g. email services and Facebook platform) and was distributed via the authors’ private and professional contacts and further spread by snowball effect. The survey was active from 12th of April until 3rd of May 2020. The participants were informed about usage and storage of data and gave consent before starting the survey. There was no compensation for participating in the study. The primary aim of the survey was to cluster changes in dietary practice, eating habits and food intake since the beginning of the first lockdown in an adult sample living in Germany. Furthermore, secondary outcomes included information on sociodemographic characteristics, anthropometrics, physical activity, health and working conditions as well as information on the pandemic’s impact on daily life to further characterize the identified clusters. The study was approved by the local ethics committee (University Duisburg-Essen, Germany, No. 20-9255-BO).

### Timeline of the first German COVID-19 lockdown

Briefly, the first German COVID-19 lockdown included the following measures ordered by the German Government at the time the survey was active (for details see daily reports of the Robert Koch Institute, Germany):

13/03/2020: closings of daycare, educational, medical and cultural institutions, sport facilities, gastronomy, and shops; telework where possible.

22/03/2020: limitation of private contacts in public spaces to two persons; social distancing.

14/04/2020: introduction of individual protection restrictions, e.g. wearing of facemasks.

20/04/2020: reopening of shops > 800 m.^2^

06/05/2020: stepwise extended reopening of shops, gastronomy, educational and medical institutions; extension of social distancing and individual protection restrictions until 05/06/2020; hot spot strategy.

### Questionnaire

The questionnaire was set up by a team of academic nutritionists and psychiatrists. The process involved several internal tests and revisions as well as a small pilot run in nine participants that revealed potential sources of inconsistencies. The final questionnaire consisted of 33 non-randomized questions and included sections on (A) changes occurring due to the pandemic concerning eating habits, body weight, and food intake, (B) physical activity, occupational status and everyday stresses, and (C) personal attitudes, general information, anthropometrics, sociodemographic factors (in mentioned order). Mean duration to finish the questionnaire was 13 min.

(A) Changes in eating habits, body weight and food intake

Participants were asked to rate changes in their eating behavior and meal aspects, e.g. time spend on food preparation, food choice motives, as well as the importance of nutrition in daily life were explored (*n* = 19 items), based on four categories (“increased considerably”, “increased slightly”, “constant”, “decreased”). Questions on eating as a means of coping with emotional stress were also included in the survey and three of them were based on the Eating Behaviour and Weight Problems Inventory (EWI) [18]. Furthermore, participants were asked to rate changes in intake of 28 food groups (for details see Fig. 3) since the beginning of the pandemic using four categories: [“more”, “constant”, “less”, “I don’t eat (this food) anyway”]. The selected food groups were roughly based on the DEGS food frequency questionnaire [19]. Changes in body weight were categorized into “increased”, “constant”, “decreased”.

(B) Changes in physical activity, occupational status, and everyday stresses

Questions on changes in leisure and sports activity were based on four categories (“increased considerably”, “increased slightly”, “constant” and “decreased”). Participants were asked about occurrences of impairment to their occupational situation and their everyday stress levels and factors (e.g. financial insecurity, childcare or relationship problems; Yes/No). Specific traits of distress like health concerns, existential fears, loneliness and boredom were explored by four categories (“increased considerably”, “increased slightly”, “constant”, “decreased”) as well as confidence in public structures as politics, health care system and public food supply (data not shown).

(III) Personal characteristics, general information, anthropometrics and sociodemographic factors

Participants were asked to classify themselves with respect to personal characteristics on a five-point Likert-type scale (“does not apply at all”, “does rather not apply”, “partially applies”, “largely applies”, “fully applies”). Six questions based on common inventories referred to impulsivity, emotional regulation (Life Problems Inventory [20]), optimism (Life Orientation Test [21]) and body satisfaction (Eating Disorder Inventory-2 [22]). Furthermore, the questionnaire collected information on age, gender, diagnosed diseases, regular medication, education level, occupational status, residency, community size, country of birth (oneself, mother, and father), and household size. Body mass index (BMI) was calculated as body weight (kg) divided by squared height (m^2^) based on self-reported data on current height (cm) and body weight (kg). BMI was categorized according to the criteria of the World Health Organization [23]. Family composition was categorized according to information on household-size and numbers of minors living in the household. Migration background was defined as having at least one parent not born in Germany [24].

### Exclusion criteria and data quality control

A total of 2310 participants finished the questionnaire. Thereof, participants aged under 18 years or with implausible information on age (*n* = 21), participants living abroad (*n* = 73) and pregnant or breastfeeding women due to differing needs (*n* = 117) were excluded. Implausible data on body weight (≤ 35 kg,  ≥ 300 kg; *n* = 14), body height (≤ 100 cm,  ≥ 1000 cm, *n* = 11) and household size (0;  ≥ 100, *n* = 7) were set to “missing” for further analysis. For the sections on changes in food intake and eating habits, a separate missing analysis was performed. In total, *n* = 109 participants had single item non-responses (< 50% missing items) in the section on food intake, and *n* = 62 participants in the section on eating habits. In accordance with the approach by Michels et al. used for food frequency questionnaires, missing data were imputed based on the assumption, that single item non-response is mainly caused by participants omitting specific items, because they are not consumed or applicable at all [25]. Therefore, omitted items were coded as “I don’t eat anyway” or “constant”, respectively. One participant was excluded due to more than 50% missing items in the section with questions on nutritional behavior. The final data set included 2103 participants.

### Identification of changes in eating habits and food intake through pattern analysis

Patterns of change were determined using two-step cluster analysis considering variables on changes in food group intake (*n* = 28 food groups), and changes in eating habits (*n* = 19 habits). Prior to analysis, response categories on food group intake and eating habits were re-categorized to create three consistent categories to better interpret the obtained patterns: “more” (included “more frequently” from food group intake and “considerably more/more often” and “slightly more/more often” from eating habits); “constant” (included “just as often” and “I don’t eat anyway” from food group intake and “just as often” from eating habits); and “less” (included “rarer” from food group intake and “less” from eating habits). Because the two-step cluster analysis is very sensitive to case order in the data set, the order was sorted using a random number. In sensitivity analysis, four further random distributions of case order were calculated, which led to comparable results in the number of clusters and their characteristics (data not shown). The number of clusters (*n* = 3) was based on the log-likelihood distance and Schwarz Bayesian criterion [26]. The cluster outputs were subjectively labeled to summarize the major changes in food intake and eating habits in each cluster, i.e. pattern of change. Cluster analysis was repeated with an internal random sample of 50% of the total study sample (*n* = 1073) and additionally with only participants who had zero missings in the sections on food intake as well as eating habits (*n* = 1949). For both sub-samples, kappa statistic was used to assess reliability of the cluster solutions. Kappa values of 0.808 for the sub-sample without missing values and 0.813 for the 50% sub-sample indicate a very good agreement.

### Statistical analysis

Categorical variables are presented as % (*n*), while continuous variables are presented as mean ± SD as they are graphically normally distributed. To identify basic factors, that influence the odds of assignment to each pattern of change compared with each other pattern, three binary logistic regression analyses were performed. A model was created, including a minimal adjustment set of potential confounders (age, gender and education), which were identified using directed acyclic graphs (DAG) [27]. DAG is a visual approach of causal assumptions to identify the presence of confounding and hence, minimal adjustment sets. Results are presented as adjusted odds ratios (aOR) and 95%-confidence intervals (95%-CI). To adjust for multiple comparisons, Bonferroni correction was applied and a *p-*value < 0.017 was considered statistically significant. All analyses were performed using SPSS version 27.0 [28].

## Results

### Participants

The analysis sample comprised 2103 people, 81% female, with a mean age of 40 (± 14) years and a mean BMI of 24.3 (± 4.4) kg/m^2^. A detailed overview on the samples characteristics is given in Table 1. Briefly, over 60% held a university degree and more than 70% were employed before the COVID pandemic. The majority lived with one additional adult or more (> 50%) or with another adult and child(ren) (28%) in medium-sized towns (24%) or cities (45%). A pre-existing somatic disease was declared by 65% of participants (e.g. allergies, metabolic disease, lung disease, etc.), 13% acknowledged a psychiatric disease.

**Table 1:** Sample characteristics according to patterns of change

Variables	Missing	Total 100% (*n* = 2103)	Patterns of change		
			Constant (*n* = 753)	Health-oriented (*n* = 770)	Emotionally- driven (*n* = 580)
Gender *%(n)*	0				
Female		80.9 (1702)	75.7 (570)	82.5 (635)	85.7 (497)
Male		18.6 (391)	23.8 (179)	17.1 (132)	13.8 (80)
Trans*		0.5 (10)	0.5 (4)	0.4 (3)	0.5 (3)
Age *mean (SD)*	0	40.4 (13.7)	43.1 (13.5)	40.7 (14.4)	36.6 (11.9)
Age categories *% (n)*	0				
< 30 years		26.6 (559)	20.1 (151)	27.5 (212)	33.8 (196)
30–39 years		26.6 (559)	25.6 (193)	24.9 (192)	30.0 (174)
40–49 years		19.2 (405)	20.1 (151)	18.1 (139)	19.8 (115)
50–59 years		17.1 (360)	20.6 (155)	17.9 (138)	11.6 (67)
≥ 60 years		10.5 (220)	13.6 (103)	11.6 (89)	4.8 (28)
BMI (kg/m^2^) *mean (SD)*	18	24.3 (4.4)	23.8 (4.2)	24.6 (4.4)	24.6 (4.6)
BMI categories^a^ *% (n)*	18				
Underweight		2.1 (44)	3.3 (25)	1.6 (12)	1.4 (8)
Normal weight		64.6 (1346)	67.8 (507)	62.2 (473)	63.9 (638)
Overweight		23.0 (480)	20.6 (154)	25.6 (195)	22.2 (128)
Obesity		10.3 (215)	8.3 (62)	10.6 (81)	12.5 (72)
Pre-existing somatic disease^b^ yes, % (*n*)	23	64.6 (1344)	65.8 (490)	64.4 (487)	63.4 (367)
Pre-existing psychiatric disease yes, % (*n*)	23	12.4 (258)	11.1 (83)	11.0 (83)	15.9 (92)
Constant prescriptives *yes, % (n)*	0	35.7 (750)	35.7 (269)	37.7 (290)	32.9 (191)
Household size *% (n*)	5				
Living alone		16.1 (338)	15.8 (119)	17.1 (131)	15.2 (88)
Single-parent family		1.8 (37)	1.5 (11)	1.4 (11)	2.6 (15)
Living with one adult (e.g. partner)		37.6 (790)	39.9 (300)	38.3 (294)	33.9 (196)
Living with at least one adult (e.g. partner) and at least one child		28.3 (594)	28.4 (214)	26.7 (205)	30.3 (175)
Living with two or more adults (e.g. flat share)		16.2 (339)	14.4 (108)	16.4 (127)	18.0 (104)
Migration background *yes, % (n)*	18	11.6 (241)	8.7 (65)	12.9 (98)	13.6 (78)
Education *% (n)*	0				
Completed university degree		61.5 (1293)	63.0 (474)	58.6 (451)	63.4 (368)
General qualification for university or technical college entrance		25.5 (537)	22.7 (171)	27.7 (213)	26.4 (153)
Secondary school certificate		12.2 (257)	13.8 (104)	12.6 (97)	9.7 (56)
No degree		0.2 (3)	0.0 (0)	0.2 (2)	0.2 (1)
Other		0.6 (13)	0.5 (4)	0.9 (7)	0.3 (2)
Pre-pandemic occupational status *yes, % (n)*	0				
Employment		71.9 (1513)	71.7 (540)	70.8 (545)	73.8 (428)
Self-employment		13.6 (285)	14.1 (106)	12.7 (98)	14.0 (81)
Seeking employment		1.2 (25)	1.6 (12)	1.0 (8)	0.9 (5)
Retirement		5.6 (118)	7.2 (54)	6.8 (52)	2.1 (12)
Maternal/parental leave		2.4 (51)	2.5 (19)	1.9 (15)	2.9 (17)
School		3.6 (75)	2.5 (19)	3.6 (28)	4.8 (28)
Other		7.0 (55)	6.0 (45)	8.4 (65)	6.6 (38)
Community size *% (n)*	0				
Rural (< 5000)		16.0 (337)	15.7 (118)	18.2 (140)	13.6 (79)
Town (5000–19,999)		16.0 (337)	17.7 (133)	14.7 (113)	15.7 (91)
Middletown (20–99,000)		23.9 (502)	25.6 (193)	23.0 (177)	22.8 (132)
City (> 100,000)		44.1 (927)	41.0 (309)	44.1 (340)	47.9 (278)

^a^Underweight (BMI < 18.5 kg/m^2^); Normal weight (BMI: 18.5 kg/m^2^ to 24.9 kg/m^2^); Overweight (BMI: 25 kg/m^2^ to 29.9 kg/m^2^); Obesity (≥ 30 kg/m^2^)

^b^Somatic disease comprises at least one of the following diagnosed diseases: lung diseases (e.g. COPD, asthma), allergies, cardiovascular diseases (e.g. heart attack, heart valve defects, angina pectoris, hypertension), cancer, autoimmune diseases (e.g. rheumatism, Hashimoto), metabolic diseases (e.g. diabetes mellitus, thyroid disease)

### Total sample: changes in eating habits and food intake

**Fig. 1:**
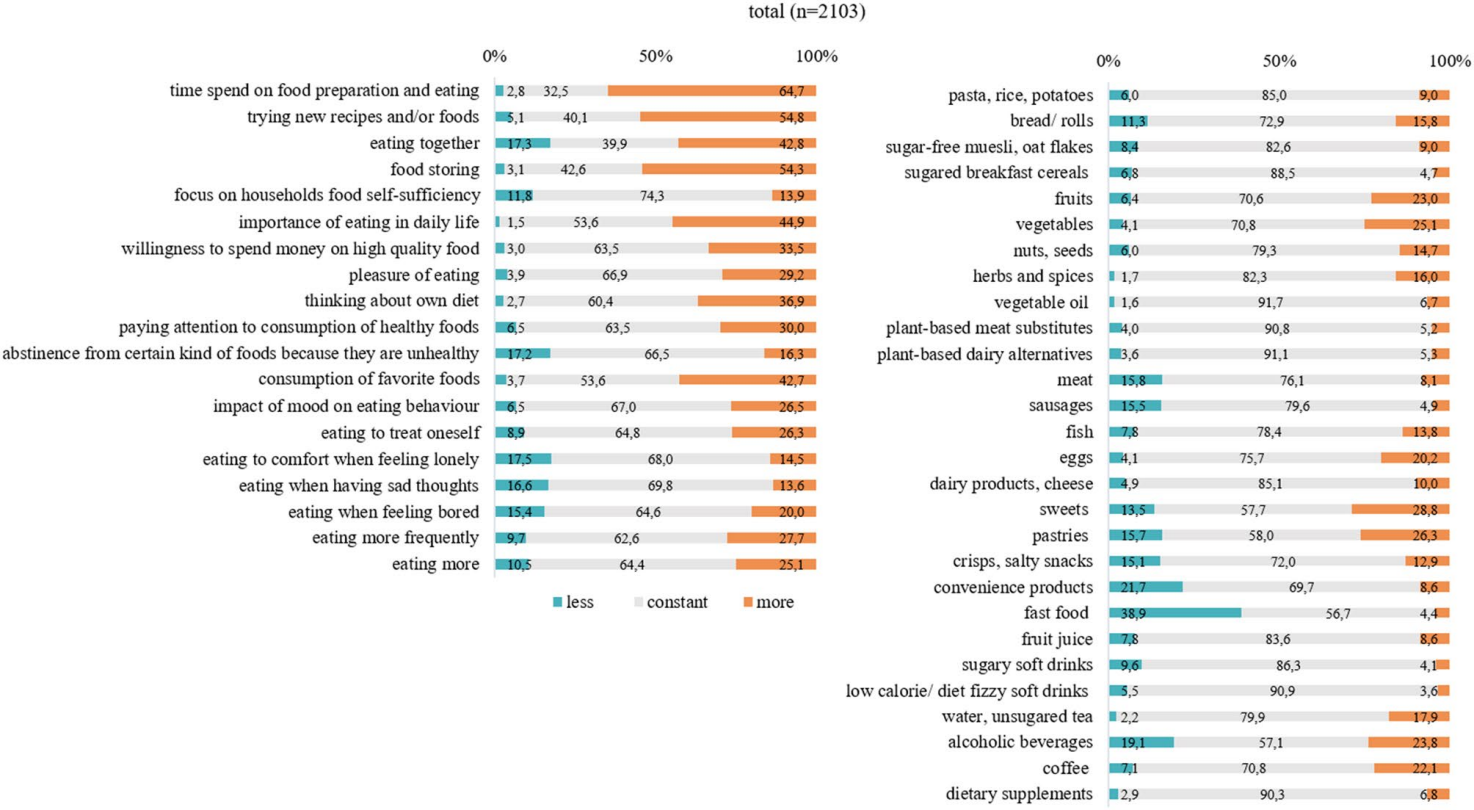
Changes in eating habits and food intake (total sample)

As shown in Fig. 1, almost 2/3 of the total sample reported spending more time with food preparation and eating and 55% experimented with new recipes and/or foods, while 43% indicated to consume their favorite foods more often. Although these circumstances come along with eating in company more often in 43% of participants, at the same time, almost 18% of the sample indicated to eat alone more often compared to pre-pandemic life. More than 40% of the total sample declared an increased importance of nutrition in their daily life, 34% an increased willingness to spend money on food and 37% increased thoughts about one’s own diet. At the level of food group intake, the documented changes were less distinct compared to changes in eating behavior. The only outstanding finding was that less fast food was consumed by almost 40% of the total sample while at least 20% indicated to eat more fruit and vegetables. We observed opposing indications for intake of sweets and pastries as well as for alcoholic beverages: 14–20% of the total sample stated to eat less, 24–29% to eat more of these food groups.

### Patterns of change in eating habits and food intake

Pattern analysis revealed three patterns of change in eating habits and food intake that characteristically differ from each other with respect to motives for food choice, eating behavior, and food intake as well as the variables of importance of eating in daily life.

#### Constant pattern (C-P)

**Fig. 2:**
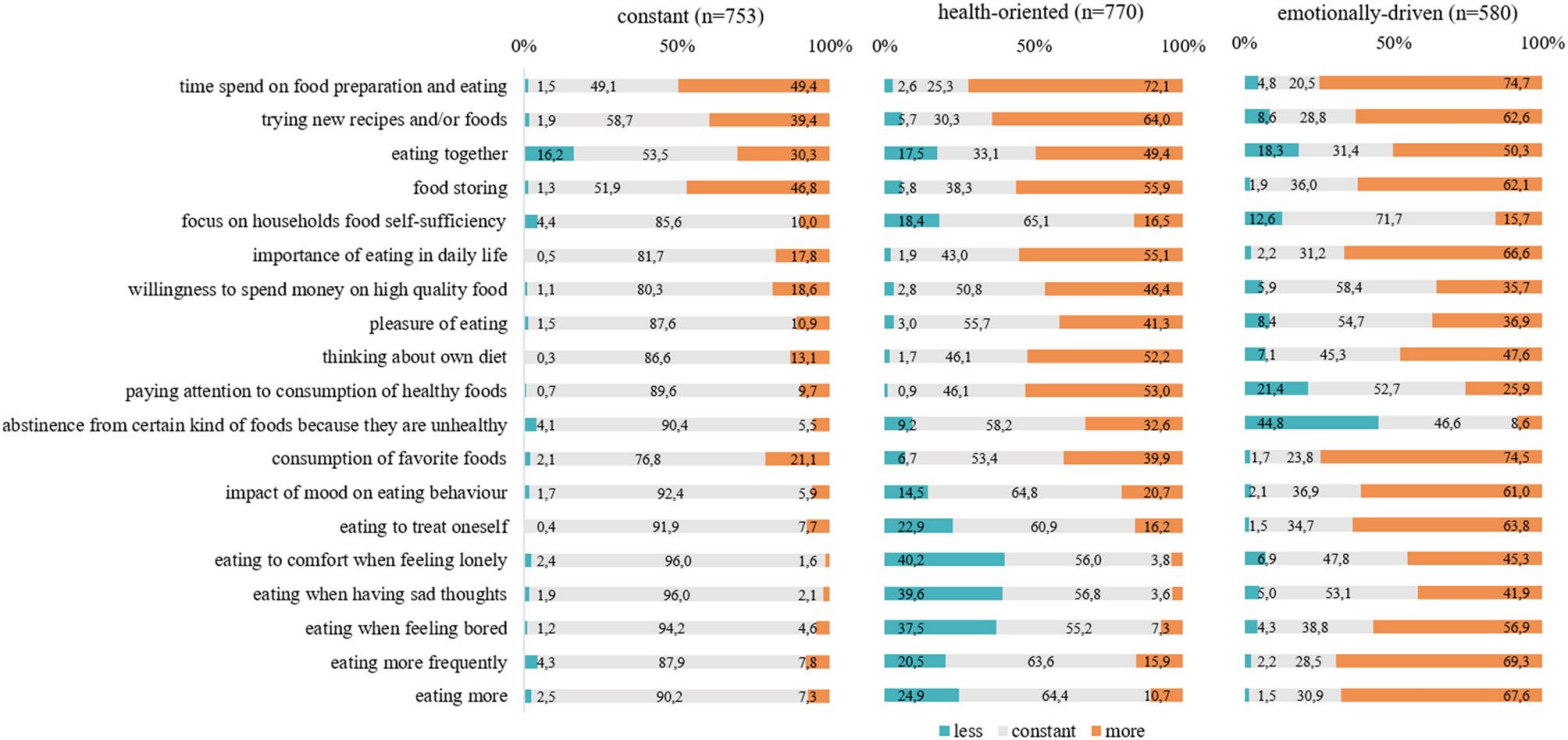
Changes in eating habits according to patterns of change

**Fig. 3:**
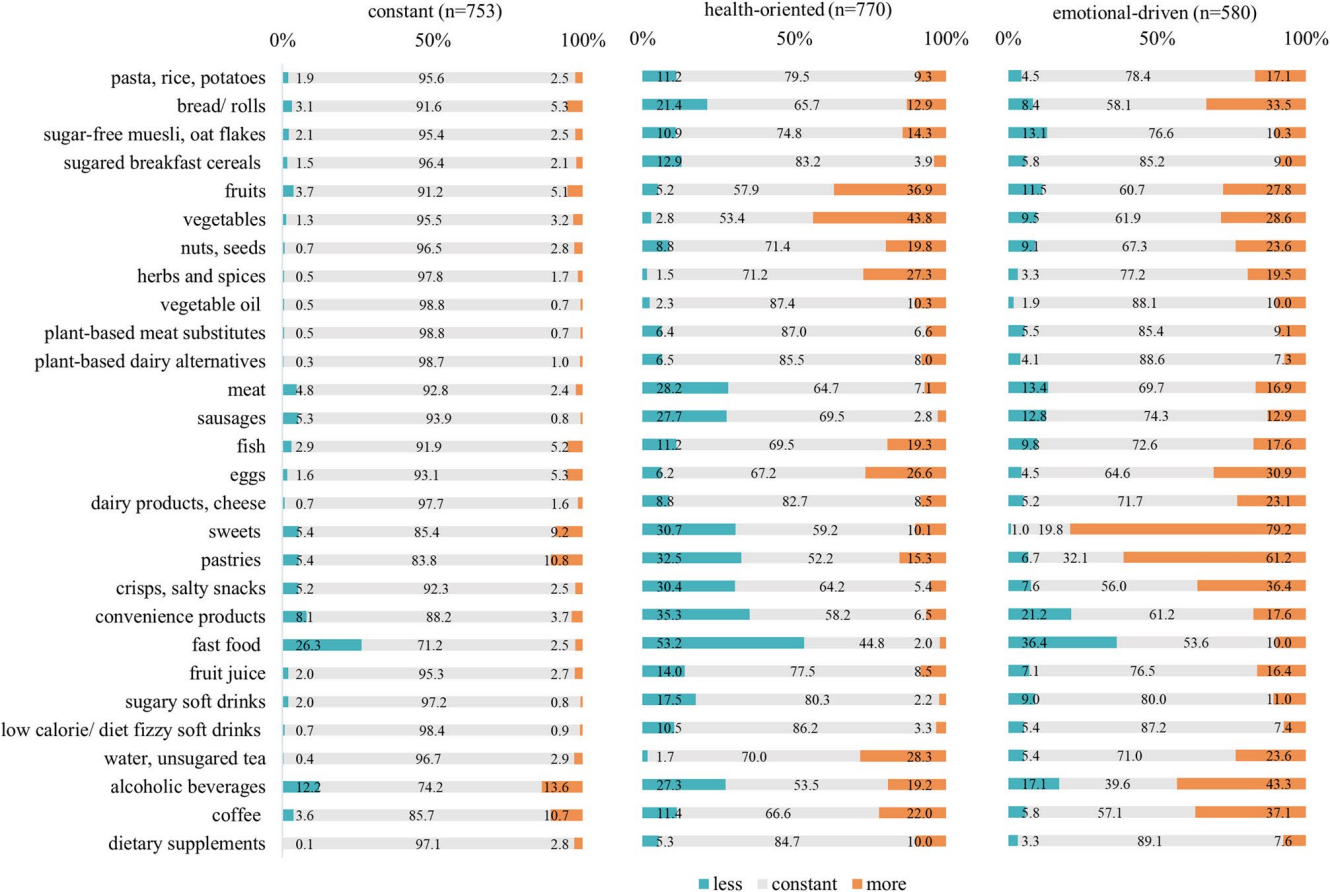
Changes in food intake according to patterns of change

A subgroup of 753 participants (36% of the sample) did not indicate any characteristic changes. The only relevant changes comprise changes in dietary practice (e.g. time spend with food preparation and eating), food storage, and fast food consumption as already described for the total sample (Figs. 2, 3).

#### Health-oriented pattern (HO-P)

In the HO-P (*N* = 770, 37%) more than 50% of the participants reported paying attention to consumption of healthy foods and 33% to refusal of certain kinds of foods because they are not healthy. They stated to eat less, less frequently and less as a reaction to emotional states (e.g. eating when feeling lonely 38%). 1/3 from the HO-P decreased their intake in sweets and pastries, more than 1/3 of the people indicated a reduced consumption of convenience products and almost 1/3 in meat and sausages (Fig. 2, 3).

#### Emotionally-driven pattern (ED-P)

Of the participants with the ED-P (*N* = 580, 28%), 75% reported an increased consumption of their favorite foods and 45% indicated less abstinence of foods considered unhealthy. Almost 70% reported to eat more, more often and more often as a reaction to negative emotional states (42–63%). More than 60% stated that their mood had now a higher impact on their eating behavior compared to pre-pandemic times. Almost 80% of the participants from the ED-P indicated an increased intake in sweets and pastries and 40% in coffee-based drinks and alcoholic beverages (Fig. 2, 3).

Compared to the C-P, the HO-P and ED-P both revealed more distinct changes concerning dietary practice and the importance of eating and nutrition: more than 70% of the allocated participants indicated to spend more time on food preparation and eating and more than 60% to testing more recipes and/or foods that are new (Fig. 3). More than 50% of the participants indicated an increased importance of eating in their daily life along with more thoughts on their diet, more willingness to spend money on food and an increased pleasure of eating (36–66%). Both patterns show a higher number of people reducing fast food consumption compared to the constant pattern (53% and 36%) as well as more people increasing their intake of fruit, vegetable, nuts, seeds, herbs and spices and eggs with the COVID pandemic (Fig. 3).

### Associations between basic factors and patterns of change

Significant associations between basic factors and patterns of change upon correction for multiple testing are presented in Table 2. Compared to the constant pattern assignment to the ED-P and the HO-P was inversely associated with male gender (aOR:0.53; 95%-CI:0.39; 0.71, aOR:0.69; 95%-CI:0.53; 0.89, respectively), and age (aOR per year: 0.96; 95%-CI:0.95; 0.97, 0.99, 95%-CI:0.98; 1.00, respectively), while positively associated with migration background (aOR:1.69; 95%-CI:1.18; 2.44, aOR:1.54; 95%-CI:1.10; 2.15, respectively), overweight (aOR:1.52; 95%-CI:1.14; 2.04, aOR:1.58; 95%-CI:1.22; 2.05, respectively) and obesity (aOR:2.46; 95%-CI:1.65; 3.67, 1.66; 95%-CI:1.15; 2.40, respectively). Additionally, assignment to the ED-P was inversely associated with underweight when compared to the C-P (aOR: 0.32; 95%-CI:0.14; 0.73), and with age when compared to the HO-P (aOR per year:0.98; 95%-CI:0.97; 0.99).

**Table 2:** Odds ratios for the association of basic factors and patterns of change

	Emotionally driven vs. constant	Health-oriented vs. constant	Emotionally driven vs. health-oriented
	aOR (95%-CI)	aOR (95%-CI)	aOR (95%-CI)
Gender			
Female	1	1	1
Male	0.53 (0.39; 0.71)*	0.69 (0.53; 0.89)*	0.83 (0.61; 1.12)
Trans*	0.79 (0.17; 3.72)	0.64 (0.14; 2.87)	1.28 (0.25; 6.67)
Age *(per year)*	0.96 (0.95; 0.97)*	0.99 (0.98; 1.00)*	0.98 (0.97; 0.99)*
BMI categories^a^			
Underweight	0.32 (0.14; 0.73)*	0.47 (0.23; 0.94)	0.78 (0.31; 1.95)
Normal weight	1	1	1
Overweight	1.52 (1.14; 2.04)*	1.58 (1.22; 2.05)*	1.00 (0.76; 1.31)
Obesity	2.46 (1.65; 3.67)*	1.66 (1.15; 2.40)*	1.45 (1.01; 2.09)
Pre-existing somatic disease	1.05 (0.83; 1.33)	1.00 (0.81; 1.24)	1.01 (0.80; 1.28)
Pre-existing psychiatric disease	1.37 (0.99;1.91)	0.92 (0.67;1.28)	1.48 (1.07;2.04)
Household size			
Living with one adult (e.g. partner)	1	1	1
Living alone	1.07 (0.76; 1.51)	1.10 (0.82; 1.48)	0.95 (0.69; 1.33)
Living with at least one adult (e.g. partner) and at least one child	1.27 (0.96; 1.78)	0.96 (0.74; 1.23)	1.25 (0.95; 1.65)
Living with two or more adults (e.g. flat share)	1.25 (0.89; 1.77)	1.13 (0.83; 1.54)	1.02 (0.74; 1.42)
Single-parent family	2.56 (1.14; 5.78)	1.06 (0.45; 2.49)	2.21 (0.99; 4.93)
Migration background	1.69 (1.18; 2.44)*	1.54 (1.10; 2.15)*	1.06 (0.77; 1.47)
Education			
Completed university degree or general qualification for university or technical college entrance	1	1	1
Lower	0.92 (0.64; 1.32)	1.03 (0.76; 1.40)	0.88 (0.62; 1.27)
Pre-pandemic occupational status			
Employment	1.00 (0.77; 1.30)	0.89 (0.71; 1.12)	1.12 (0.88; 1.44)
Self-employment	1.29 (0.92; 1.79)	0.97 (0.72; 1.31)	1.27 (0.91; 1.76)
Retirement	0.72 (0.35; 1.47)	1.53 (0.96; 2.45)	0.50 (0.24; 1.02)
Community size			
City (> 100,000)	1	1	1
Middletown (20–99,000)	0.86 (0.64; 1.14)	0.84 (0.65; 1.09)	1.00 (0.76; 1.33)
Town (5000–19,999)	0.84 (0.61; 1.16)	0.77 (0.57; 1.04)	1.01 (0.73; 1.40)
Rural (< 5000)	0.74 (0.52; 1.04)	1.07 (0.79; 1.43)	0.70 (0.50; 0.96)

Adjusted for age, gender and education (aOR), with 95% confidence interval (95% CI)

^*^Significant results upon Bonferroni correction for 3 comparisons between clusters, i.e. *p* < (0.05/3 = 0.017)

^a^Underweight (BMI < 18.5 kg/m^2^); Normal weight (BMI: 18.5 kg/m^2^ to 24.9 kg/m^2^); Overweight (BMI: 25 kg/m^2^ to 29.9 kg/m^2^); Obesity (≥ 30 kg/m^2^)

### Changes in body weight and physical activity

#### Body weight

**Fig. 4:**
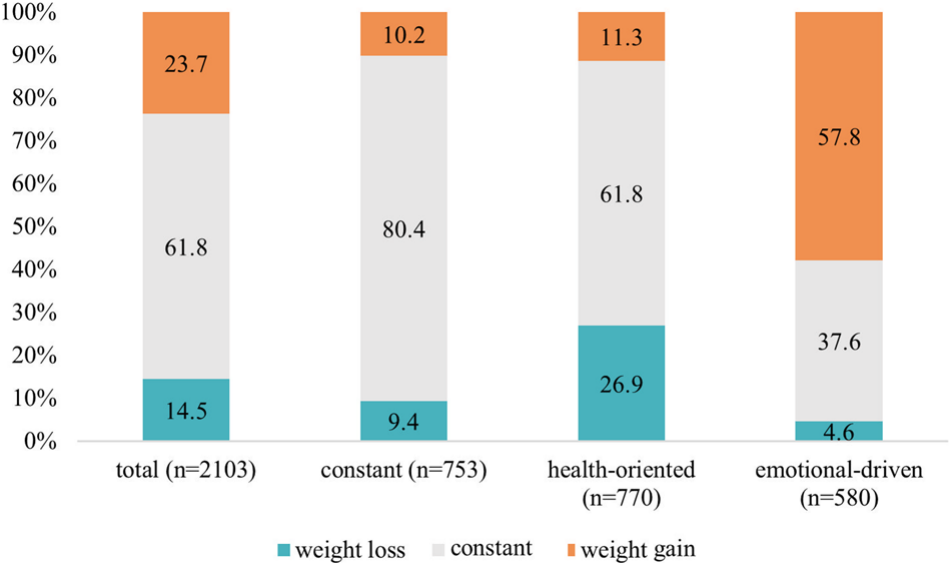
Changes in body weight

During the pandemic, 24% of the total sample reported to have gained and 15% to have lost weight. In the C-P, 81% of the participants reported no change in body weight, whereas 10% indicated a loss and 10% a gain in body weight, respectively. In contrast, 27% in the HO-P indicated weight loss, whereas 58% in the ED-P reported weight gain (Fig. 4).

#### Physical activity

**Fig. 5:**
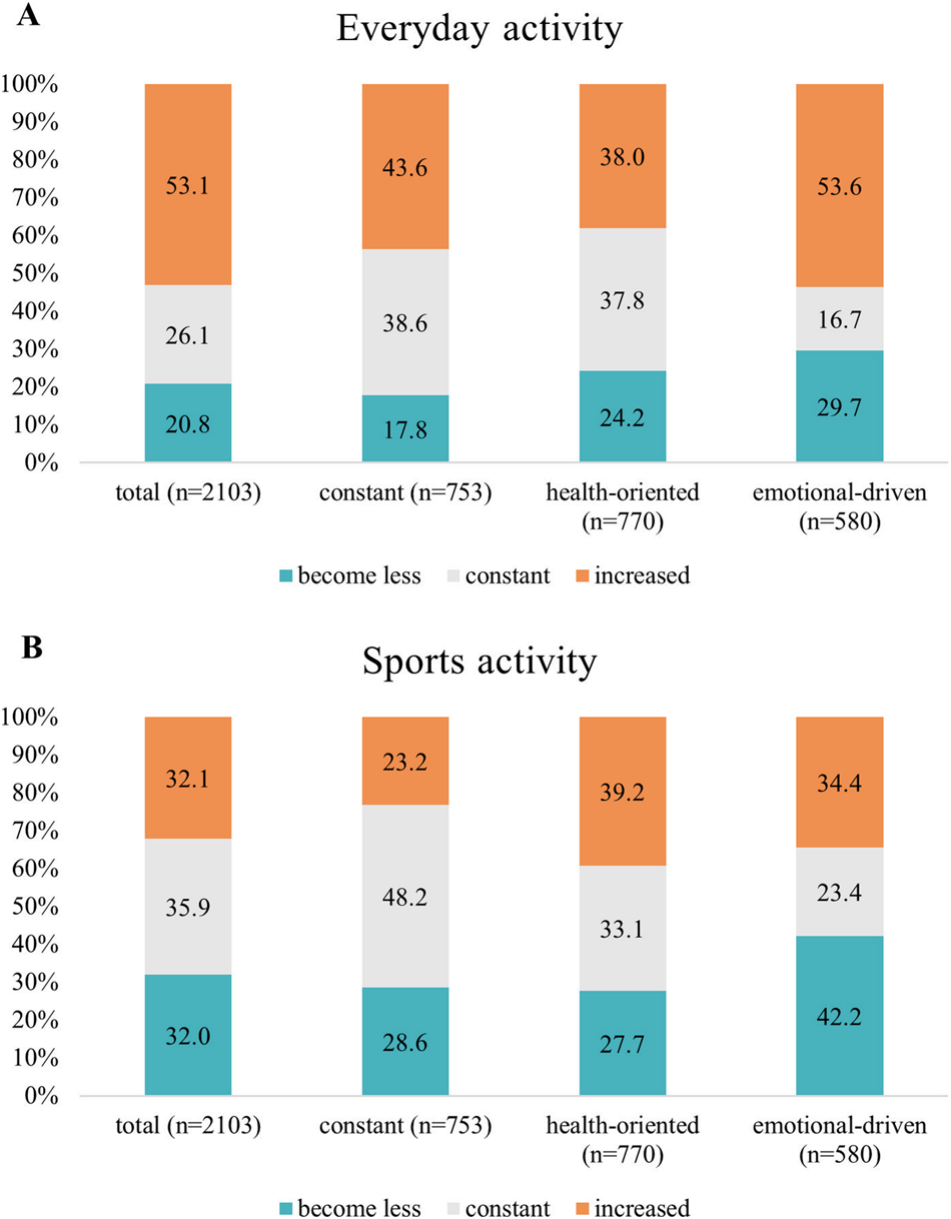
a/b Changes in everyday activity (**a**) and sports activity (**b**)

Everyday activity increased during the pandemic in more than 50% of the total sample and decreased in 21%. Participants in the ED-P contributed the highest proportion of participants with increased everyday activity (ED-P: 54%, C-P: 44%; HO-P: 38%) (Fig. 5a). Changes in sports activity were almost equally distributed to constant/less/more in the total sample. The HO- and the ED-P each contributed higher proportions of participants with increased sports activity (HO-P: 39%; ED-P: 34%, C-P: 23%) then the C-P. The ED-P also revealed high proportions of participants with less sports activity (ED-P: 42%, C-P: 29%, HO-P: 28%) (Fig. 5b).

## Discussion

Within the presented pattern analyses, we used an approach to integrate changes in eating habits and food intake as a consequence of the COVID-19 pandemic, considering data from a large-scaled population-based online survey in Germany. The sample was predominantly composed of young to middle-aged women, with the majority being urban residents, highly educated, working, without children, but living in joint households.

Overall, almost 2/3 of the total sample stated to be affected in their usual dietary practice and reported intensified food preparation and storage. With respect to more specific traits of eating habits and food intake, cluster analysis revealed three characteristic patterns of change: the *constant pattern* (C-P, 36%) was only slightly affected by the pandemic. Both other patterns share several changes, but differ distinctively with regard to changes in food choice motives and intake of specific food groups, i.e. a *health-oriented (*HO-P*,* 37%) and an *emotionally-driven pattern* (ED-P). The latter represented the smallest group (28%). Regression analyses revealed that in this study population the odds of changing eating habits and food intake either to HO-P or ED-P were higher in women, people with migration background, younger participants, and increased with BMI categories (based on self-report). Additionally, of those who changed their pattern, especially younger people moved towards the ED-P. The findings should be interpreted with respect to the study’s limitations, as described beneath.

### General differences between the patterns of change

In the *constant pattern* (C-P), representing 1/3 of our sample, we observed only changes that can be regarded as an expected consequence of closure of gastronomic services, staff restaurants/canteens and delivery services (more time with food/meal preparation and eating, variance in recipes, less fast food) and/or as consequence of initial feelings of nutritional insecurity (more food storage). We assume that this pattern is at least partly composed of people experiencing only little disruptions of their daily life.

Compared to C-P, these fundamental aspects of dietary practice were influenced in markedly higher proportions in the HO-/ED-P. Both patterns share some further developments: participants reported an increased importance of nutrition in their daily life, that was also reflected by growing attention to quality of foods and willingness to pay. More participants indicated increased enjoyment of food, diet patterns where opted by reduced fast food consumption in favor of more health-promoting food items like fruit and vegetables, nuts, seeds, fish and eggs as well as herbs and spices, the latter typifying a more creative and varying diet. This trend of (although not solely) favorable changes in diet patterns under lockdown conditions has also been described by previous studies [29, 30] and is associated with financial means, food availability, and nutrition knowledge [31, 32]. For both, HO-P and ED-P, we assume an interaction of public closures, withdrawal into private spheres and home cooking to increase wellbeing and enjoyment of health-promoting food items: In pre-pandemic times, 40% of European adults claimed a “busy lifestyle” as a barrier to healthy eating. This barrier was significantly associated with less cooking at home and less vegetable intake but high intake of fast food [33]. During the COVID pandemic, many people in Germany had extra time due to short-time work or less commuting time due to teleworking. As opposed to C-P, we presume home cooking and social meals were not only an unavoidable necessity for many participants in the HO-/ED-P, but also enriched lockdown routines by relief from boredom and possibilities for self-realization. This may not only add more healthy foods to dietary patterns but also help to psychologically adapt as qualitative studies imply [34]. Including positive emotions such as joy, happiness and their influence on eating habits into the survey, would have complemented the current assumptions, as these in particular have been associated with more openness to experiences and hedonic pleasures [35].

### Specific characteristics of health-oriented and emotionally-driven pattern

However, beside these similarities both patterns show unique characteristics and add to previous studies from Germany that reported opposing trends within the study populations with regard to changes in lifestyle, food intake and, thus, body weight [36, 37]. The *health-oriented pattern* (HO-P) is less homogenous in its pattern of change than C-P and ED-P. It, however, distinguishes from both other patterns with a higher fraction of those who stated to deliberately consume more healthy foods, and to abstain from foods they considered unhealthy. The most distinct difference to the ED-P is expressed by increased abstinence from sweets, snacks, fast food, convenience and alcoholic beverages as well as less eating as a response to negative affect, lower eating frequencies and smaller amounts. A reduction in meat (products) in favor of plant-based foods is apparent, although less pronounced. Almost 40% of the HO-P reported higher ambitions in sport activities and, 1/3 documented body weight reduction. Although differing in methodological details, the essence of the HO-P appears very similar to the “favorable change” pattern of the French NutriNet-Santé cohort (20%) [31], also characterized by a healthier dietary pattern, increased activity and decreased body weight. Almost 90% of this pattern stated that the confinement period was a good opportunity to balance their diet and to deliberately have focused on weight reduction. Notably, we did not assess potential engagements in body weight reduction in our study. Considering that a BMI above “normal” increased the odds of changing to the HO-P with the pandemic, however, it seems reasonable that the HO-P deliberately engaged to body weight reduction during lockdown measures because they might have experienced less barriers then, e.g. less hectic daily routines and external eating cues. On the other hand, we cannot exclude that the HO-P includes people with unhealthy extents of dieting behaviors. The prevalence of eating disorders rose since the very early beginning of the pandemic all over the world [38–42] and increased restrictive eating behaviors have been observed not only among those with a pre-existing eating disorder, but also in the general population (28%) [43]. We did not assess dietary restraint/ restrictive eating per se, but 40% of the HO-P indicated more abstinence from foods regarded as being unhealthy and less eating in order to cope with negative feelings than before the pandemic. Both behavioral traits may also appear with unhealthy extents of rigid avoidance [44], fixated either on weight control or on healthy eating. Thus, although the described dietary changes appear favorable in general, this pattern of change may also include participants who possess adverse dieting behaviors.

The *emotionally-driven pattern* conversely is characterized by a high proportion of participants that indicate overeating due to negative emotions, e.g. higher frequencies of eating, higher amounts in foods eaten and increased effects of mood on eating behavior. Along with that higher intakes of sweet and fatty foods and alcoholic beverages were reported. Comparable findings have been described from countries all over the world [45–47] and also within the NutriNet-Santé cohort a pattern with increased body weight, sedentary time and eating due to negative affect was described (“unfavorable changes”, 37%) [31]. Considering that fractions of the ED-P also documented higher enjoyment of foods and less rigidity in food choices (e.g. less abstinence of foods considered unhealthy) one may speculate that a subgroup favored hedonic pleasures and the comforting effect of food over cognitive control on their food choices. As essential parts of wellbeing were eliminated by measures of social distancing (e.g. company, autonomy, participation) and negative affect increased [6], focusing on pleasure-seeking and hedonic aspects of life may have contributed to re-balance wellbeing. To the best of our knowledge, there is no evidence on the relationship of emotional eating and subjective wellbeing, but we speculate that a certain degree of emotional eating can be perceived as consistent within a troubling experience of an early world-wide pandemic In this regard, assessment of positive emotions and their influence on eating habits would contribute to clarification and differentiation of motivations for the observed overeating in the ED-P, but the presented study does not allow for further explanation. The proposed narrative, however, would not be true for at least half of the declared “emotional eaters” in this pattern (emotional eating: 60–70% vs. higher enjoyment of foods: 35%). Moreover, previous analyses suggest that the higher odds for overweight and obesity in the ED-P hint to dispositions to susceptibility to food intake and emotional eating as well as maladaptive coping strategies, [45] and higher negative emotional eating has also been associated with binge eating [48]. Furthermore, the ED-P showed the highest proportions of people that indicated a gain in body weight which is of concern for public health and preparation for future pandemics. Respective strategies should involve support of craving control, as replicated findings from UK’s and Australia’s first lockdown show that this behavioral trait is an important predictor for intake of energy dense, sweet or salty snacks (“comfort foods”), thereby predisposing individuals to weight gain [16, 17].

In line with previous studies on dietary changes during the pandemic we found younger people and women to be more affected in their dietary practice [30, 31, 37, 49], either changing to HO- or ED-P. Females not only show a stronger affectation on mental health by lockdown measures [6, 50–52] but also may be more susceptible to changes in eating behavior [53]. Explanations for the influence of age are manifold and include discrepancies in impairments of daily and thus, dietary routines as well as differing degrees of mental stability and extents of mental impairment by pandemic and lockdown. Younger people have probably been more affected in their daily and dietary routines by e.g. closing of university canteens, discontinuance of social life or multiple responsibilities (e.g. care work, teaching, working) while older people exhibit more established routines and behaviors. Furthermore, older people appear more stable and/or less affected in overall mental health with the pandemic [36, 49, 52, 54] which again may disturb eating habits less compared to younger people. Furthermore, we found different proportions of patterns for people with migration background and higher odds of changing their eating habits to ED-/HO-P. Due to the small sample size of people with migration background and no further existing national evidence on this association, this finding must be treated with caution. However, representative national studies show higher scores for depression and anxiety due to the pandemic in people with migration background, presumably explained by e.g. worries about relatives living abroad, stronger impairment by travelling restrictions [50, 55]. The stronger mental impairment may predispose people with migration background to changes in eating habits.

The presented findings as well as most other studies in this context merely show patterns at a given time point. To evaluate the relevance of the evidence on changes in eating behavior and food intake with a pandemic and respective lockdown measures, longitudinal analyses constitute important mainstays in public health research. First results indicate that in some individuals, eating behaviors changed during the course of the first lockdown to progressively increasing or decreasing food intakes (UK, March to June 2020) [56]. Furthermore, comparative findings from lockdowns surveys in Australia hint to higher restrictive eating behavior and lower quality of life in the 2^nd^ wave lockdown [57].

### Strength and limitations

Our findings benefit from a large sample of a rather homogenous population. The two-step cluster analysis offered the identification of different reactions to the pandemic and public lockdown. Moreover, the integrative approach considered both, eating habits and food intake, thereby showing a broader picture of overall nutrition. Accordingly, we were able to add some aspects of wellbeing (e.g. pleasure of eating) to a rather health-dominated perspective on dietary practice. However, the study has several limitations. *Study sample.* As discussed earlier, the main limitation is that the study sample was not representative and selection bias is very likely as we assume that people interested in nutrition are largely overrepresented, while ethnicities other but Germans without migration background, people with lower socioeconomic background, and men are underrepresented. Accordingly, proportions and details of the patterns have limited external validity. This point is strengthened by differing proportions compared to the patterns found within the representative NutriNet-Santé study [31]. *Assessment.* All findings were based on single retrospective assessments, self-ratings and no attention check was applied, making them prone to reporting and recall bias. However, our questionnaire assessed the time frame since the beginning of the lockdown, i.e. 6–7 weeks at maximum. This is substantially shorter than the usual time frame e.g. in food frequency questionnaires and reduces the risk of recall bias. *Questionnaire design.* With respect to a rather exploratory design, the survey comprised a broad selection of traits related to eating and dietary practice to the detriment of specificity in single traits of eating behavior. The questionnaire was not experimentally validated ahead because it was impossible to predict the duration of the lockdown in Germany and we decided to start the study immediately. However, we used questions from established instruments as basis for our questions as far as possible and applied a small pretest to identify internal inconsistencies. *Cluster analysis.* Labelling of the derived pattern, as common in data driven pattern analyses, was subjective.

Future studies investigating dietary reactions to stressful life events should combine repeated assessment of food intake with commonly used measures of specific eating behavior traits to further characterize different reactions in eating habits, e.g. restrictive eating, negative and positive emotional eating, impulsive eating. Certainly, it would be crucial to access ethnic disparities and to survey more vulnerable populations, e.g. people who are at risk for mental health problems and/or are low in income, to have a solid base for risk assessment and, i.e. support across settings.

## Conclusion

In a sample of predominantly highly educated, young to middle-aged women without children, from shared households, nearly two of three people changed their dietary practice during the first German COVID wave/ lockdown. Those with more intense changes can be divided in one cluster that deliberately balanced their diet to a healthier, potentially rigid pattern and one cluster whose eating behavior was largely formed by hedonic pleasures and emotional needs. Both patterns of change may contribute to recover from the potentially troubling experience of the pandemic but they also reveal that eating habits may be relevantly disturbed in subgroups, thereby increasing the risk of eating disorders and overweight. This is of special interest if early adverse changes in habits manifest during the course of the pandemic. Results from this study indicate that people being responsive to topics of health and nutrition (e.g. females, young age, people with high body weight) presumably are more likely to report distinct changes. Understanding further trajectories of the patterns may be helpful for evaluation of risk and protection factors.

## Data Availability

Raw data and SPSS Syntax are available from the corresponding author on reasonable request.
